# Large-scale localization of touching somas from 3D images using density-peak clustering

**DOI:** 10.1186/s12859-016-1252-x

**Published:** 2016-09-15

**Authors:** Shenghua Cheng, Tingwei Quan, Xiaomao Liu, Shaoqun Zeng

**Affiliations:** 1 School of Mathematics and Statistics, Huazhong University of Science and Technology, 1037 Luoyu Rd, Building of Science - 715, Wuhan, 430074 China; 2 Britton Chance Center for Biomedical Photonics, Huazhong University of Science and Technology-Wuhan National Laboratory for Optoelectronics, Wuhan, 430074 China; 3 MoE Key Laboratory for Biomedical Photonics, Department of Biomedical Engineering, Huazhong University of Science and Technology, Wuhan, 430074 China; 4 School of Mathematics and Statistics, Hubei University of Education, Wuhan, 430205 China

**Keywords:** Touching soma localization, Density-peak clustering, Optical microscopic image

## Abstract

**Background:**

Soma localization is an important step in computational neuroscience to map neuronal circuits. However, locating somas from large-scale and complicated datasets is challenging. The challenges primarily originate from the dense distribution of somas, the diversity of soma sizes and the inhomogeneity of image contrast.

**Results:**

We proposed a novel localization method based on density-peak clustering. In this method, we introduced two quantities (the local density ρ of each voxel and its minimum distance δ from voxels of higher density) to describe the soma imaging signal, and developed an automatic algorithm to identify the soma positions from the feature space (ρ, δ). Compared with other methods focused on high local density, our method allowed the soma center to be characterized by high local density and large minimum distance. The simulation results indicated that our method had a strong ability to locate the densely positioned somas and strong robustness of the key parameter for the localization. From the analysis of the experimental datasets, we demonstrated that our method was effective at locating somas from large-scale and complicated datasets, and was superior to current state-of-the-art methods for the localization of densely positioned somas.

**Conclusions:**

Our method effectively located somas from large-scale and complicated datasets. Furthermore, we demonstrated the strong robustness of the key parameter for the localization and its effectiveness at a low signal-to-noise ratio (SNR) level. Thus, the method provides an effective tool for the neuroscience community to quantify the spatial distribution of neurons and the morphologies of somas.

**Electronic supplementary material:**

The online version of this article (doi:10.1186/s12859-016-1252-x) contains supplementary material, which is available to authorized users.

## Background

Reconstructing brain-wide wiring networks at single-neuron resolution is the key to understanding how neuronal circuits orchestrate complex behaviors [1], and represents a major engineer challenge [2–4]. To achieve this goal, many computational techniques are required such as the localization and segmentation of neuronal somas, which is the first step in digital neuronal circuit reconstruction. Soma segmentation can provide the spatial distribution and morphometrics of somas, which are quantitative aspects of some brain disease diagnosis [5, 6]. For example, in Alzheimer's disease we can sometimes observe selective loss of nigral neurons [7], whereas in the cortices of patients with Huntington disease there is an increase in the density of large glia and a reduction in the neuronal size and density [8]. Recent advances in molecular labeling [9, 10] and imaging techniques [11–16] have enabled imaging of the whole mouse brain at a micron spatial resolution, and have provided a database for the mapping of neuronal circuits. However, the localization and segmentation of neuronal somas from this type of dataset is challenging. These challenges primarily originate from the following three aspects: the dense distribution of somas (touching somas), the diversity of soma sizes, and the inhomogeneity of image contrast.

Many methods have been proposed for the automatic localization of touching cells, such as watershed algorithms [17–20], graph-based methods [21–25], energy functional-based models [26–31] and machine learning approaches [32–34]. Additionally, some special methods are available for splitting touching cells [35–42], such as distance transform based cell detection [35], concave point-based segmentation methods [36, 37] and the gradient flow tracking method [38, 39]. These methods have their own advantages and behave well for some specific applications. However, most require enhancement for wider applications. For example, watershed algorithms often suffer from over-segmentation of cells when the image contrast is inhomogeneous; initial localizations are not easy to set using energy functional-based models to locate touching cells; distance transform based cell detection method faces challenges for the case that multiple cells touching each other; and gradient flow tracking often results in under-segmentation in locating touching cells. Therefore, these methods experience difficulties in locating touching somas from large-scale 3D images in which a dense distribution of somas, diversity of soma sizes and inhomogeneity of image contrast are common. Recently, two methods have been proposed for the large-scale localization of neuronal somas. The first is our previous method named NeuroGPS [43], which introduces the regularization item in the sphere fitting model to eliminate the influence of the thick neurites on soma localization. However, when using NeuroGPS to locate closely positioned somas, the accuracy depends on the reasonable assignment of initial positions (seeds). Assigning too many initial positions usually generates false positive positions. The second method use mean-shift clustering to search soma positions (i.e., the center point of the cluster) [44], which generates high recall and precision rates for the analysis of specific datasets. However, the number of clusters in mean-shift clustering is determined by the key parameter, kernel width. A big kernel width usually leads to a small number of clusters, and conversely a large number of clusters is for small kernel width. Thus, it may be difficult to find a reasonable kernel width that is suitable for the diversity of soma sizes.

Here, we proposed a method for the automatic large-scale localization of neuronal somas. This method was based on density-peak clustering [45], in which two quantities (the local density ρ of each voxel and its minimum distance δ from voxels of higher density) were introduced and formed the feature space (ρ, δ). From the feature space, we developed an automatic algorithm to find the clusters. Each cluster corresponds to the morphology of a soma, which achieves the localization of a soma. We demonstrated the validity of the proposed method for the large-scale localization of somas. We also demonstrated its strong anti-noise ability, the robustness of the key parameter for localization, and the high efficiency in the analysis. Furthermore, we tested our method on two image stacks. From one dataset, our method achieved the localization with a *F*_1_-measure of 0.93, which was far superior to some state-of-the-art algorithms. From the other large scale dataset (4.3 × 2.4 × 2.7 mm^3^, 3.26 GB), our method located approximately 35,000 somas and achieved *F*_1_-measures of 0.93 and 0.98 from the analysis of two sub image stacks.

## Methods

### Data acquisition

The experimental datasets were obtained by imaging a mouse brain with the two-photon fluorescence micro-optical sectioning tomography system (2p-fMOST) [16]. All experiments were performed in compliance with the guidance of the Experimental Animal Ethics Committee at Huazhong University of Science and Technology. The original size of the volume pixel was 0.5 × 0.5 × 2 μm^3^; it was merged to 2 × 2 × 2 μm^3^ for our analysis. We used three experimental image stacks in Figs. 4c, 5 and 6. The synthetic data consisted of 28 image stacks with different signal-to-noise ratios (SNR = 1, 2, 4 and 6). Each image stack contained only one pair of somas. The synthetic data were used in Fig. 3 and 4a, b. In addition, we tested the proposed method on Nissl staining datasets [46] and structured illumination microscopy datasets [47] (Table 1).

**Table 1:** Performance comparison of FarSight, NeuroGPS, HeY’s method, GFT and the proposed algorithm in different datasets

Dataset	Type	Volume	Ground truth	Proposed	FarSight	NeuroGPS	HeY’s method	GFT
Data 1	2p-fMOST	150 × 150 × 150	788	764/0.92/	805/0.85/	767/0.78/	620/0.70/	490/0.60/
		2 × 2 × 2 μm^3^		0.95/0.93^a^	0.83/0.84	0.80/0.79	0.89/0.78	0.97/0.74
Data 2	2p-fMOST	100 × 100 × 100	288	280/0.92/	268/0.75/	265/0.84/	246/0.77/	231/0.74/
		2 × 2 × 2 μm^3^		0.95/0.93	0.81/0.78	0.92/0.88	0.90/0.83	0.92/0.82
Data 3	2p-fMOST	100 × 100 × 100	25	24/0.96/	24/0.88/	24/0.96/	24/0.96/	24/0.96/
		2 × 2 × 2 μm^3^		1.00/0.98	0.92/0.90	1.00/0.98	1.00/0.98	1.00/0.98
Data 4	2p-fMOST	100 × 100 × 100	164	170/0.94/	179/0.76/	172/0.88/	165/0.82/	144/0.73/
		2 × 2 × 2 μm^3^		0.91/0.92	0.70/0.73	0.84/0.86	0.81/0.81	0.83/0.77
Data 5	2p-fMOST	100 × 100 × 100	148	147/0.96/	173/0.86/	146/0.94/	147/0.84/	135/0.86/
		2 × 2 × 2 μm^3^		0.97/0.96	0.74/0.80	0.95/0.95	0.85/0.85	0.94/0.90
Data 6	SIM^b^	100 × 100 × 100	496	486/0.95/	528/0.82/	407/0.82/	399/0.79/	452/0.89/
		1 × 1 × 2 μm^3^		0.97/0.96	0.77/0.80	0.99/0.90	0.99/0.88	0.98/0.93
Data 7	SIM	100 × 100 × 100	93	89/0.96/	97/0.88/	84/0.90/	87/0.90/	83/0.88/
		1 × 1 × 2 μm^3^		1.00/0.98	0.85/0.86	1.00/0.95	0.97/0.93	0.99/0.93
Data 8	Nissl staining	100 × 100 × 100	695	616/0.85/	680/0.86/	551/0.77/	502/0.66/	501/0.71/
		1 × 1 × 1 μm^3^		0.95/0.90	0.84/0.85	0.97/0.86	0.91/0.77	0.98/0.82
Data 9	Nissl staining	100 × 100 × 50	287	266/0.90/	263/0.79/	216/0.74/	197/0.67/	187/0.64/
		1 × 1 × 1 μm^3^		0.97/0.93	0.86/0.82	0.98/0.84	0.97/0.79	0.99/0.78
Mean ± SD			/recall	0.93 ± 0.04	0.83 ± 0.05	0.85 ± 0.08	0.79 ± 0.10	0.78 ± 0.12
			/precision	0.96 ± 0.03	0.81 ± 0.07	0.94 ± 0.07	0.92 ± 0.07	0.96 ± 0.05
			/*F*_1_	0.94 ± 0.03	0.82 ± 0.05	0.89 ± 0.06	0.85 ± 0.07	0.85 ± 0.08
two-side *p*-value of Mann–Whitney test				/recall	0.001^**^	0.021^ns^	0.005^**^	0.008^**^
compared with the proposed method				/precision	0.000^**^	0.893^ns^	0.209^ns^	0.789^ns^
				/*F*_1_	0.000^**^	0.051^ns^	0.010^**^	0.025^*^

*n*s not significant

*p ≤ 0.05, ** *p* ≤ 0.01

^a^Number of detected cells/recall/precision/*F*_1_-measure

^b^Structured illumination microscopy

**Fig. 1:**
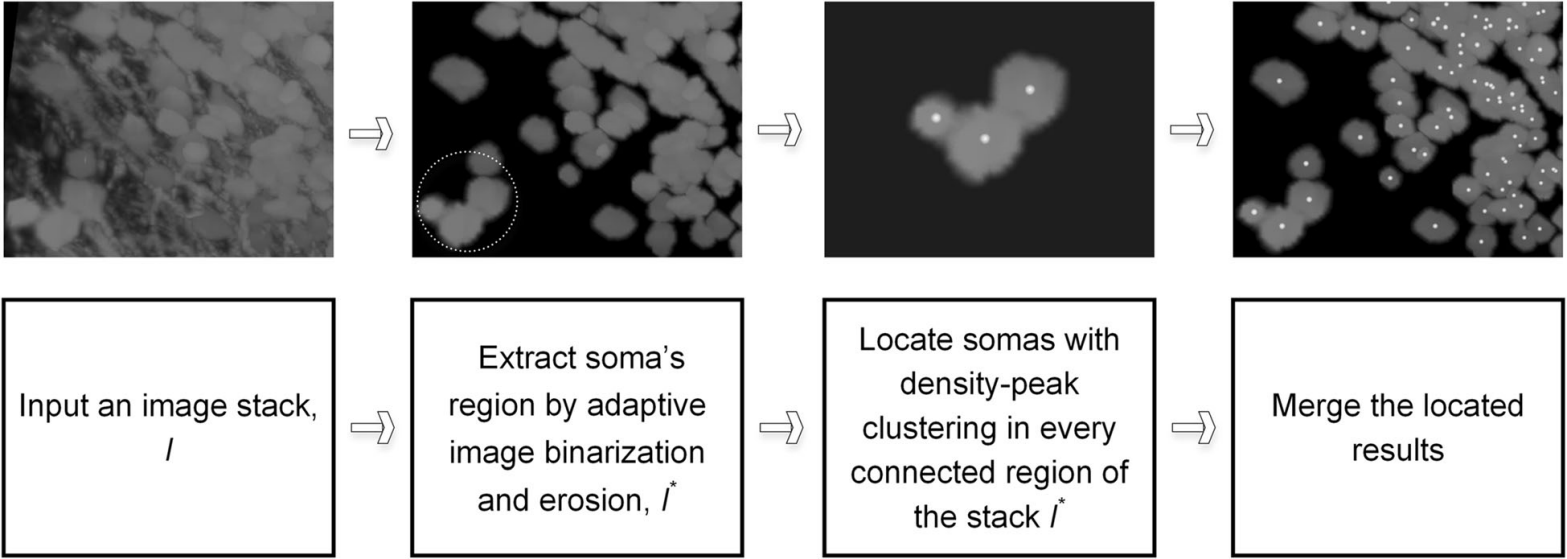
The flow chart of the proposed method for the localization of neuronal somas

Our proposed method for the location of the neuronal somas consists of the following three steps: 1) extract the soma’s region by using an adaptive image binarization and erosion procedure; 2) locate somas with modified density-peak clustering; and 3) merge the located results. Detailed descriptions of each step are provided in the following sections. We also depict the entire routine of the proposed method in Fig. 1. Notably, our method can also segment the soma’s shape, and we present the related segmentation procedure.

### Estimation of the soma’s region

The procedure for estimating the soma’s region consists of the following steps: 1) split the image stack into sub-blocks; 2) binarize each sub-block and erode each binarized sub-block; 3) merge all eroded sub-blocks into a single image stack; and 4) extract the connected region from the merged image stack. We regard the extracted region as the soma’s region in which one or several somas may be included.

Generally, the signal intensities of somas vastly change in large-scale image stacks. This phenomenon increases the task difficulty in distinguishing between the foreground and background. Considering this point, we split a large-scale image stack into sub-blocks and analyze these sub blocks instead of the whole image stack. Using this procedure, we reduce the range of the signal intensity. When splitting the image stack, the size of a sub-block is set to approximately 200 × 200 × 200 voxels. Any two neighboring regions have overlapping regions to eliminate the boundary effects. The overlapped width is set to 12 fold of the size of a voxel (24 μm). This setting can eliminate boundary effects and avoids the extra calculations required for the overlapped regions.

For each sub-block, let *I*(*x*, *y*, *z*_*k*_) be its *k*th slice. We binarize *I*(*x*, *y*, *z*_*k*_) for all *k*, with the following formula

1 $$ B\left(x,y,{z}_k\right)=\left\{\begin{array}{l}1\kern1em I\left(x,y,{z}_k\right)>C\left(x,y,{z}_k\right)+thr{e}_{\mathrm{binarization}}\sqrt{C\left(x,y,{z}_k\right)}\\ {}0\kern1em \mathrm{otherwise}\end{array}\right. $$

Here, *C*(*x*, *y*, *z*_*k*_) represents the background image, generated as follows: the image min (*I*(*x*, *y*, *z*_*k*_), *thre*_OTSU_) is convolved 10 times with averaging template of 3 × 3 × 1 pixels. Here, 3 × 3 × 1 and 10 are empirical values that can ensure that the convolved images are sufficiently smooth and approach the background. *thre*_OTSU_ is a binarization threshold estimated by Otsu’s method [48]. *thre*_binarization_ in Eq. (1) is the predetermined binarization parameter. The above binarization procedure is based on the assumption that *C*(*x*, *y*, *z*_*k*_) can be approached with the Poisson background model, which is suitable for most images collected with optical microscopy. The selection principle of *thre*_binarization_ should assure that the soma regions can be completely identified, and allows the identified region to contain a small part of the background points. Generally, *thre*_binarization_ is set to larger values 5 – 8 if the signal intensity of the soma regions is more than double the intensity of the background; otherwise it is set to 2 – 4. Based on this principle, *thre*_binarization_ is set to 2 for the simulation dataset in our analysis. This value is low and ensure that all soma regions can be identified (even for SNR = 1) and that the identified region contain only about 3 % of the background points, estimated with the Poisson background model. For experimental signals with high SNRs but with soma artifacts, *thre*_binarization_ is set to 6 to eliminate the soma artifacts. Notably, the soma regions consist of voxel points with *B*(*x*, *y*, *z*_*k*_) = 1.

The binarized image stack *B* also contains artifacts and noise points. Therefore, we eliminate these unnecessary points using the erosion operation [49]. For each volume pixel with a value of 1, we set its value to zero when the sum value of this volume pixel and its 26-connected volume pixels is less than the erosion threshold *T*; otherwise, there is no change in its value. We perform this operation for all this type of volume pixels in image stack *B* to complete one erosion operation. We iteratively repeat this erosion operation until the eroded image stack reaches steady state. We set the erosion threshold *T* to 9 in the first erosion operation and continuously increase this value with a step of 0.027 for the subsequent erosions. The maximum value of *T* is less than 11. The specific stability condition of erosion refers to that the relative change rates of two indices, generated with the erosion operation, are less than the given threshold *thre*_erosion_ (0.1 % in our experiment). In these two indices, one is the number of voxels with value 1 and the other is the number of connected regions in the eroded image. Here, *thre*_erosion_ and *T* are used to control the intensity of erosion. The reasonable number of erosion operations should ensure that the soma morphologies cannot be damaged and is determined by the voxel size and the minimum radius of the cells. We verified that the settings in our experiment are suitable for voxel sizes ranging from 1 × 1 × 1 μm^3^ to 2 × 2 × 2 μm^3^ and soma radii ranging from 3 to 10 μm.

We merge all eroded image stacks into a single image stack using the following method. We detect the overlapped region of two neighboring sub-blocks according to the label information from the splitting of the image stack. The overlapped region contacts two un-overlapped regions and is equally divided into two regions denoted by *R*_*a*_ and *R*_*b*_. This partition leads to one sub-block denoted by *A* with *R*_*a*_ and *R*_*b*_ indicating the interior and boundary regions, respectively, and the other denoted by *B* with *R*_*b*_ and *R*_*a*_ indicating the interior and boundary regions, respectively. We assign the signal of *A* in region *R*_*a*_ and the signal of *B* in region *R*_*b*_ to the overlapped region. We merge all neighboring sub-blocks in *x*-axis direction, thereby making the size of the merged sub-blocks the same as the size of the original image stack in the *x*-axis direction. By continuously using the same operation in the *y*- and *z*-axis directions, all sub-blocks can finally be merged into a single image stack.

Finally, we extract the connected region (which refers to the estimated soma region) from the merged image stack using region growing. During the extraction, two volume pixels with values of one are regarded as connected if their positions appear in the sphere region with the radius of the square root of 3. Next, we locate somas in each connected region.

### Localization and segmentation of somas based on fast search of density peaks

For each connected region, we use the density peak clustering method [45] to locate and segment somas. Briefly, we locate somas by finding density peaks of the signal (cluster center) and segment somas via the cluster assignment.

The density peak clustering is recently proposed clustering method. It depends on two quantities: the local density ρ_*i*_ of each point, and its minimum distance δ_*i*_ from points of higher density. The two quantities construct the 2D feature space (ρ, δ). The feature space, combined with the hypothesis “cluster center is characterized by a higher density ρ than their neighbors and by a relatively large distance δ” [45], provides the effective information for data clustering. However, the original density-peak clustering method has a disadvantage in that it requires the manual selection of cluster centers. It is infeasible to select cluster centers manually when we use the method to locate thousands of cells from large-scale images. Therefore, we developed a new method to automatically select cluster centers.

**Fig. 2:**
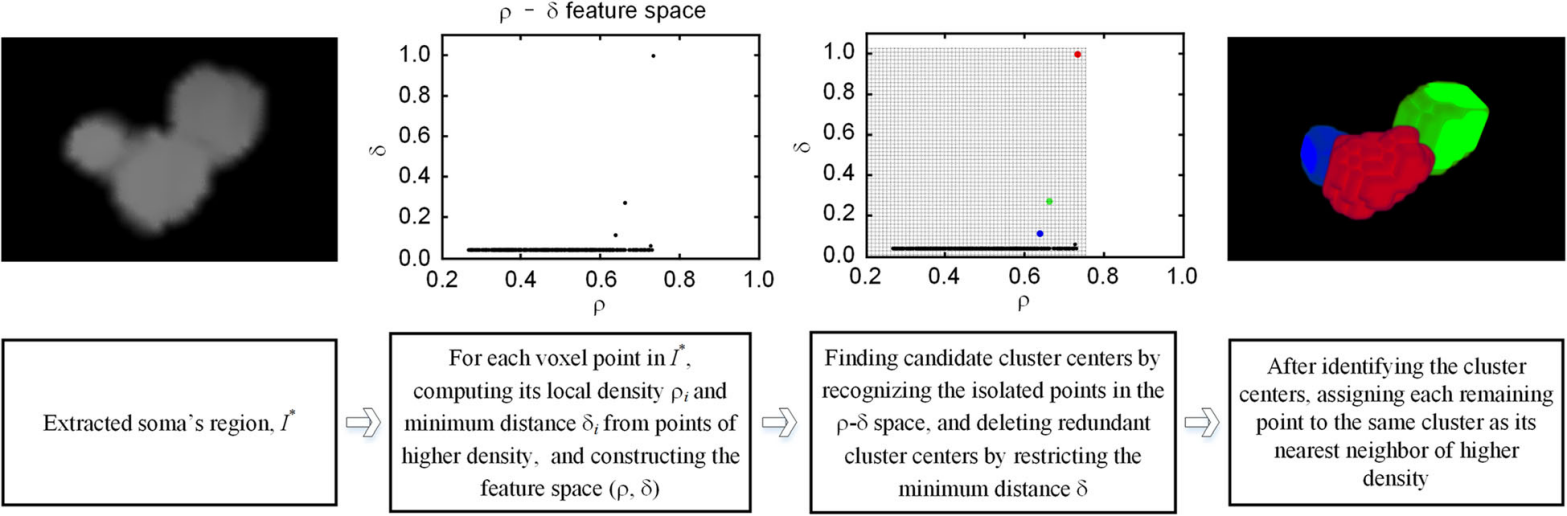
The steps for the localization and segmentation of somas

The modified density peak clustering method applied for soma localization and segmentation consists of three parts (Fig. 2):

- computing the local density ρ_*i*_ of each voxel point, and its minimum distance δ_*i*_ from points of higher density, and constructing the feature space (ρ, δ);
- finding candidate cluster centers by recognizing the isolated points in the ρ-δ space, and deleting redundant cluster centers by restricting the minimum distance δ;
- assigning cluster for the points except the identified cluster centers.

#### Computing ρ, δ

For the voxel point *p*_*i*_ (*i* = 1, 2, …, *m*) in the estimated soma region, the local density is defined as

2 $$ {\rho}_i=\frac{1}{Z}{\displaystyle \sum_{j:\;{\left\Vert {p}_i-{p}_j\right\Vert}_2\le R}I}\left({p}_j\right)K\left({p}_i,\kern0.5em {p}_j\right)=\frac{1}{Z}{\displaystyle \sum_{j:\;{\left\Vert {p}_i-{p}_j\right\Vert}_2\le R}I}\left({p}_j\right)\frac{1}{\sqrt{2\pi}\upsigma} \exp \left(-\frac{\left|\right|{p}_i-{p}_j{\left|\right|}_2^2}{2{\upsigma}^2}\right) $$

Here, *I*(*p*_*i*_) represents the signal value of volume point *p*_*i*_, *K*(*p*_*i*_, *p*_*j*_) is a Gaussian kernel function with a kernel width σ, *Z* is a normalization constant, *R* is the window radius of the kernel function (*R* = 2σ), and ||.||_2_ is 2-norm. In our experimental dataset analysis, the kernel width σ is set to 4 μm, which is slightly more than half of the average value of the soma’s radius.

After obtaining the local density of each voxel point, we calculate the minimum distance δ of a voxel point using the following formula

3 $$ {\updelta}_i=\left\{\begin{array}{l}\frac{\underset{j:\kern0.5em {\uprho}_j>{\uprho}_i}{ \min }{\left\Vert {p}_i-{p}_j\right\Vert}_2}{\underset{\forall i,j}{ \max }{\left\Vert {p}_i-{p}_j\right\Vert}_2}\kern1.6em {\uprho}_i<\underset{\forall j}{ \max }{\uprho}_j\kern1em \\ {}1\kern7.50em {\uprho}_i=\underset{\forall j}{ \max }{\uprho}_j\end{array}\right. $$

#### Identifying cluster centers

Cluster centers are characterized by a higher density ρ than their neighbors and by a relatively large distance δ, and act as isolated points in the ρ-δ space. Therefore, the possible cluster center points can be selected according to the feature density Λ (the density computed in the ρ-δ space). The redundant cluster centers can be removed by restricting the minimum distance δ. Below are the specific steps.

Step 1) *Discretize the feature space*. We equally divide the intervals (0, max ρ_*i*_) and (0, max δ_*i*_) into the [1000max ρ_*i*_] + 1 and [1000max δ_*i*_] + 1 subintervals, respectively. Here, [.] is a rounding operation. We count the number of feature points (ρ, δ) that drop into the grids to generate a feature image. We convolve the generated image with a two-dimensional Gaussian window, and obtain the filtered image that contains the information of the density of the feature points. Here, the size of the two-dimensional Gaussian window is 11 × 11 grids and the kernel widths for *x*- and *y*-coordinates are both set to 3-fold the size of the grid.
Step 2) *Estimate the density of the feature points*. For each feature point, we estimate its density by using the value of the pixel, whose region contains the feature point, in the filtered image. This density is named the feature density, denoted by Λ.
Step 3) *Generate the candidate cluster center points*. We obtain the cluster center points by using the following formula
  4 $$ \left\{{p}_i{\left|\;\varLambda (i)\le thre\right.}_{\mathrm{selective}}\kern0.24em \&\kern0.62em \updelta (i)\ge \frac{R_{\min }}{\underset{\forall i,j}{ \max }{\left\Vert {p}_i-{p}_j\right\Vert}_2}\right\} $$
  Here, *thre*_selective_ is a predetermined parameter and is set to 10^-2^ in our analysis. *R*_min_ is the minimum value of the estimated soma radius, and is approximately equal to 3 μm in our dataset. Notably, we usually set *thre*_selective_ to a small value to ensure that the candidate points contain all soma positions in the estimated region. The redundant points generated here can be deleted in Step 4.
Step 4) *Delete redundant cluster centers*. We sort the candidate center points generated in *Step 3* in the order of the descending signal density ρ, obtained with Eq. (2). We traverse the points in this order using the following method. For the current point, we search its nearest point among the points that have not been traversed, and calculate the distance between the point and its nearest point. If the distance is less than the minimum value of the estimated soma radius, we label this nearest point. The above procedure is repeated until all candidate points are traversed. We regard the unlabeled candidate points as the final cluster centers, i.e., the positions of somas.

#### Assigning cluster

After identifying the cluster centers, we assign each remaining point to the same cluster as its nearest neighbor of higher density. The detailed steps are described below.

Step 1) *Label the cluster centers*. We assign a unique sequence number to each cluster center.
Step 2) *Sort the points in the estimated region*. We sort the points in the estimated region in the order of the descending local density ρ. The sorted points exclude cluster centers.
Step 3) *Assign the points to their clusters*. For the current sorted point, we search its nearest point among the points that have higher local densities ρ than the current point. If the nearest point has been assigned to a sequence number, we assign this number to the current point; otherwise, no operation is performed on this point. Using this way, we traverse all sorted points and the unassigned points form a new point series.
Step 4) *Repeat Step 2* for the new point series until all points are assigned to their clusters. Points with the same labels are placed into the same cluster.

### Performance evaluation

We use the precision rate, recall rate and *F*_1_-measure to evaluate the localization results derived by the algorithms. We regard the manually localized positions as true positions. We define an automatically localized position as a true positive position provided that the distance between the automated localized and true positions is less than the fixed threshold, which is set to 8 μm in our experiment. The precision rate is defined as the ratio of the true positive positions to the automatically localized positions. The recall rate is defined as the ratio of the true positive positions to the manually localized positions. The *F*_1_-measure is defined as $\frac{2\times precision\times recall}{precision+ recall}$. Note that these three evaluation indices are influenced by the threshold used to identify the true positive positions. The reasonable value should ensure that the evaluation indices change slowly when increasing or decreasing this threshold around the pre-set value.

## Results

### Segmentation of simulated touching somas at different levels of SNR

**Fig. 3:**
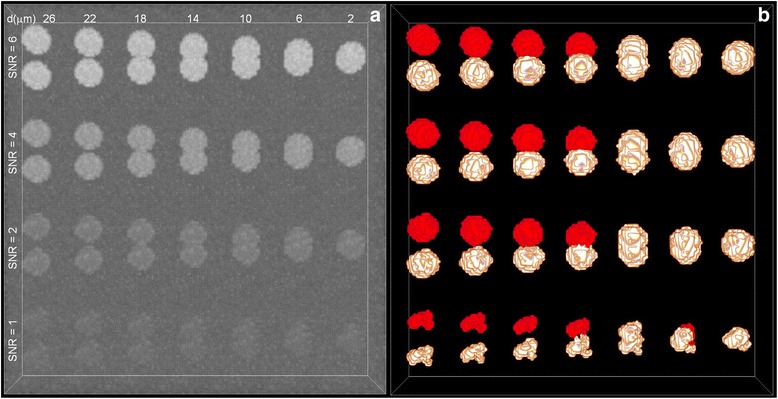
Segmentation of touching somas at different levels of SNR. **a** The simulation datasets that contain 28 pairs of somas. All somas had the fixed radius of 10 μm and the distance of a pair of somas, denoted by *d*, ranged from 2 μm to 26 μm. **b** The segmented results on the simulated datasets derived from our method

A simulation test was performed to validate the effectiveness of our method for soma localization. The synthetic data consisted of 28 image stacks with different signal-to-noise ratios (SNR = 1, 2, 4 and 6). Each image stack contained only one pair of somas. At each SNR level, there were 7 image stacks with different levels of overlap. All somas had a fixed radius of 10 μm, and the distance between a pair of somas (denoted by *d*) ranges from 2 to 26 μm. When generating the simulation dataset, let the sphere represent a soma. The signal in the inner and out region of a soma is a Poisson signal with mean value of *I*_o_ + *I*_b_ and *I*_b_. SNR is defined as the ratio of *I*_o_ to the square root of *I*_o_ + *I*_b_. *I*_b_ was fixed and set to 100 in our analysis. In this case, the SNR was determined by *I*_o_. All simulation datasets are shown in Fig. 3a, and their localization results are shown in Fig. 3b. From Fig. 3b, we see that our method effectively separated the severely-touching somas at low SNR levels (*d* = 14 for SNR = 1). Additionally, the separated somas, the slightly-touching somas, and the severely-touching somas were well located and segmented with our method if the SNR was equal to 2 or greater than 2. These results indicate the effectiveness of our method.

### Robustness of clustering parameter on soma localization

**Fig. 4:**
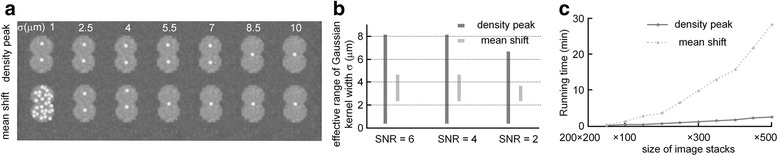
Comparison of density-peak clustering and mean shift in parameter robustness and computation complexities when segmenting touching somas. **a** Localization results of simulated touching somas with different values for σ in the condition of SNR = 3, *d* = 14 μm. **b** Effective range of σ for the two methods with SNR = 2, 4 and 6. **c** Running time (excluding the time of image preprocessing) of the two approaches on mouse hippocampal image stacks of different sizes

Our method used density-peak clustering for the localization of a soma. Compared with published localization methods [44, 50, 51] that employ mean-shift clustering [52] for this purpose, our method had a stronger robustness of the clustering parameter on soma localization. We used the simulated datasets, generated with the above-described procedure, to verify this point. These two cluster methods both have a common parameter (kernel width) that influences the localization results. Generally, a large kernel width results in a smooth signal point density curve, but loses the soma boundary information and thus easily confuses densely positioned somas. A small kernel width retains most of the soma boundary information but leads to more than one density peak in the inner region of a soma. Therefore, we regarded kernel width as the key cluster parameter, and we quantified the influence of kernel width on the localization results (Fig. 4a, b). We compared the localization results derived using these two methods on one dataset (Fig. 4a). The results indicated that the reasonable value of kernel width for density-peak clustering ranged from 1 to 7 μm, which was far larger than the range (2.5 - 4 μm) used for mean-shift clustering. We used more datasets to verify this conclusion and obtained the similar results (Fig. 4b), although the reasonable range narrowed as the SNR decreased.

Additionally, we compared the computation efficiencies of these two methods. We used an experimental image block from the hippocampal region with a 200 × 200 × 500 size to generate the testing datasets, which consisted of the first 50, 100, …, 500 slices of this image stack. The results indicated that our method had linear time complexity and was approximately 10-foldfaster than mean-shift based localization method when analyzing a large scale dataset with more than 400 slices (Fig. 4c). The localization part of our method included three steps: computing the local density ρ; searching the minimum distance δ; and identifying cluster center points. The bottleneck was the search for the minimum distance. We reduced the searching space to speed up our method using two strategies: locating somas in every connected region rather than in the whole image stack and using local search for the minimum distance in each connected region. We demonstrated that the complexity of our method is about proportional to the volume of the image stack. The detailed demonstration of the algorithm complexity is provided in Additional file 1.

### Localization of the touching somas from an experimental dataset

**Fig. 5:**
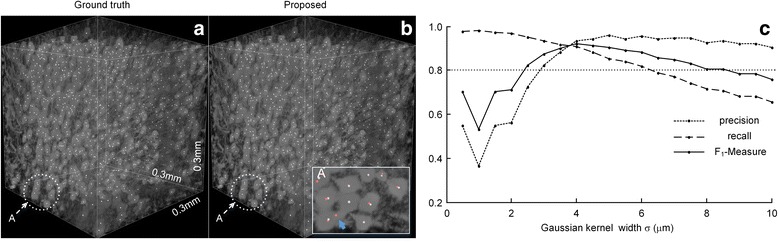
Soma localization results derived by the proposed method. **a** The manual localizations from the image stack with the total size of 150 × 150 × 150 voxels and with the voxel size of 2 × 2 × 2 μm^3^. **b** Shows the localization of the somas derived by the proposed approach. The detailed results can be found in the enlargement of the region (circles). White dots indicate manually located positions of somas; red dots are the positions located by our method. Arrows and triangles indicate the false positive positions and the missing positions respectively. **c** Robustness of Gaussian kernel width on the localization. The evaluation indexes, including localization precision, recall and *F*_1_-measure, are obtained using our method to analyze this dataset and are plotted against kernel width

We used a dataset with closely positioned somas to validate the effectiveness of our method for touching soma localization. The dataset was from a hippocampal region with a volume of 150 × 150 × 150 (2 × 2 × 2 μm^3^). From this dataset, 788 somas were manually detected (Fig. 5a) and many somas touched one another. We presented the soma localizations derived by the manual method and our method in Figs. 5a and b. Our method located 764 somas. The recall and precision rates were 0.92 and 0.95, respectively. The somas on the boundary were neglected in the quantifications, which could explain why some somas were not manually labeled. Furthermore, our method also behaved well when the key parameter, kernel width, vastly changed. Figure 5c showed that our method provided the segmented results with *F*_1_ scores greater than 0.8 for kernel width ranging from 2.5 to 8.0 μm. These two boundary values, 2.5 and 8 μm, vastly deviated from the optimal parameter of 4.0 μm, representing slightly more than half of the average soma radius. This result indirectly verified that our method was suitable for dealing with the diversity of soma sizes. From the above results, we conclude that our method is effective at locating touching somas.

### Large-scale soma localization

**Fig. 6:**
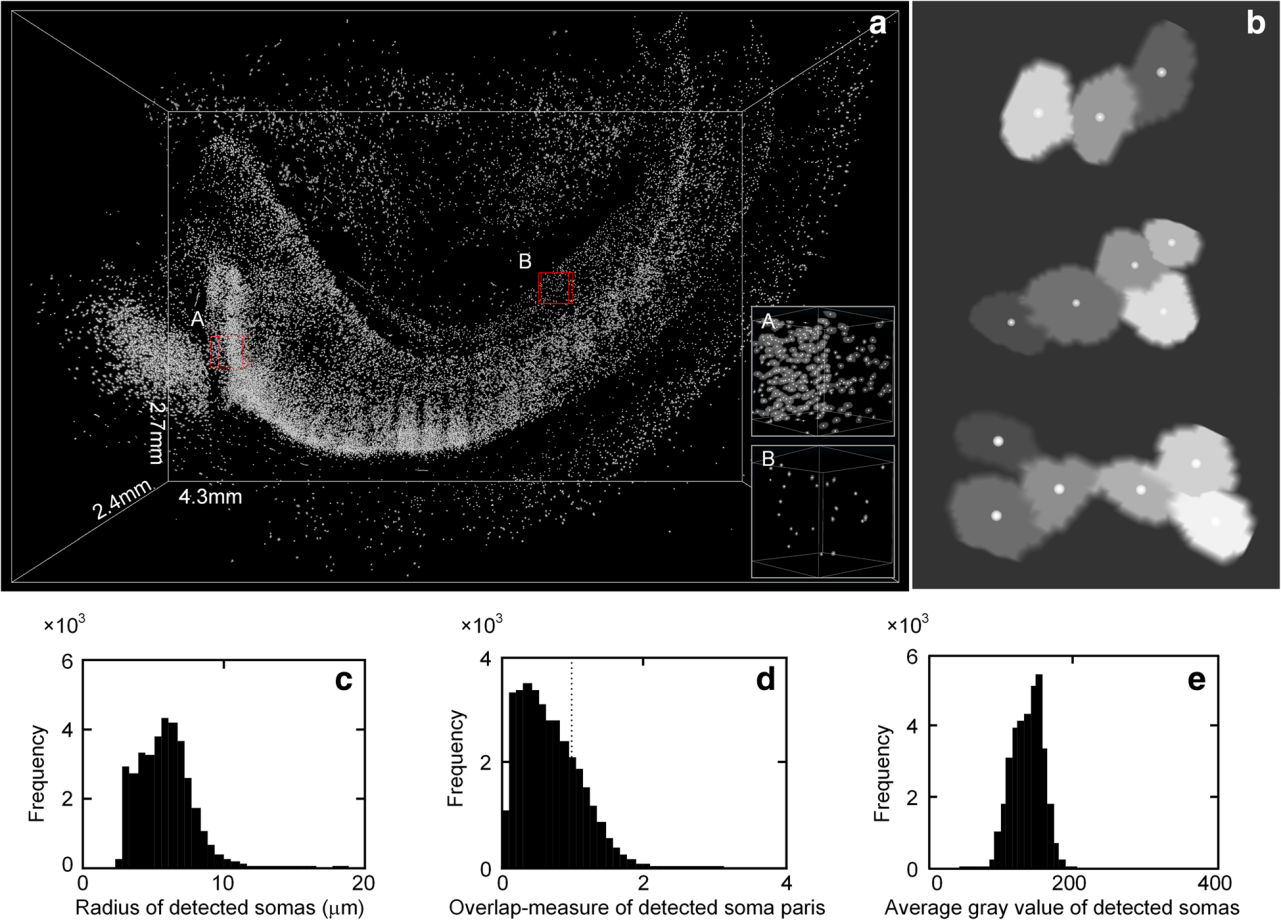
Localization of somas from large-scale dataset. **a** The max-intensity projection of an mouse hippocampal image stack with a volume of 4.3 × 2.7 × 2.4 mm^3^ and the detected soma centers (white dots). Sub-figures A and B indicate the localizations of densely positioned somas and sparsely positioned somas. **b** The localization and segmentation of touching somas in three connected components. Note that different gray level here represents different somas. **c**-**e** The frequency histograms of radius, overlap-measure and average gray value of detected somas. (radius: average distance between perimeter points of a soma and its center; overlap-measure, for a soma pair that consists of a soma and its closest soma, let *s* and *d* denote the sum of radius of these two somas and the distance between the two soma’s centers, overlap-measure is defined as the ratio of *s* to *d*; average gray value: average gray value of all points in a soma.)

Our method also effectively located somas from huge and complicated datasets. We used a dataset in which somas exhibited diversity in their spatial distributions and sizes to test our method. The size of this dataset was 2124 × 1200 × 1370 and the voxel size was 2 × 2 × 2 μm^3^. A total of 35274 somas were detected when our method was used to analyze this dataset. To quantify the segmentation, we selected two typical subregions with the same size of 100 × 100 × 100, labeled with *A* and *B*. Region *A* contained densely positioned somas and region *B* contained sparsely positioned somas, as shown in Fig. 6a- *a* and *b*. The quantified results were as follows: for region *A*, the recall rate, precision rate, and *F*_1_-measure were 0.95, 0.92, and 0.93, respectively, whereas for region *B*, these three quintiles were 1.00, 0.96, and 0.98, respectively. This result indicates the validity of our methods on this dataset. Figure 6b shows the touching soma segmentation results of three connected regions. Note that the different gray levels here represent different somas. We also illustrated the complexity of this dataset by quantifying related quantities. In Fig. 6c, we calculated the radii of the detected somas and the corresponding radius distribution with a mean of 5.9 μm and a standard deviation of 1.8 μm. Figure 6c showed that the radii of the detected somas were in a wide range (primarily 3 to 10 μm), indicating the diversity of soma sizes. In Fig. 6d, we calculated the distribution of the overlap measure of the detected somas. The overlap measure refers to the ratio of the total radii of a pair of detected somas to the distance between this pair of somas. A detected soma and its closest detected soma form a pair of detected somas. From this definition, an overlap measure of more than 1 corresponds to the two somas touching one another. In Fig. 6d, this value of more than 1 accounted for 23 % of the somas, indicating a certain number of touching measures. Figure 6e showed the different levels of signal intensity of the detected somas, which primarily ranged from 80 to 200. These statistical results verify the complexity of the dataset, which may be challenging for the previous methods.

### Comparison of the proposed method with other methods in different datasets

We compared our method with four other methods (FarSight [42], our previously proposed method NeuroGPS [43], HeY’s concave point-based method [37] and the gradient flow tracking (GFT) [38]) and quantified the localizations obtained with these five methods on nine different experimental datasets. These datasets were from three types of images: 2p-fMOST [16], Nissl staining [46], structured illumination microscopy [47]. We made Mann-Whitney [53] test of evaluation indexes (precision, recall, *F*_1_-measure), since they were not normal distributions, and analyzed the test results.

The localization evaluation indexes of these methods on the nine datasets were listed in Table 1. The last two rows at the table were values of mean, standard deviation and the results of Mann-Whitney test. For the recall index, the *p*-values were all less than 0.05, indicating the proposed method is superior to the other approaches in recall. This was because the other methods easily generated under-segmentation for densely positioned cells (Data 1, Data 2, Data 4, Data 6, Data 8, Data 9), resulting in lower values of recall. For sparsely positioned cells (Data 3, Data 5, Data 7), almost all methods could get fine results. Though the proposed method was not significantly better than the other approaches in precision (except FarSight, *p*-value, 0.000), it still kept high accuracy (mean value, 0.96) and was more stable than other methods (standard deviation, 0.03 vs. ~0.07). The test results of *F*_1_-measure also indicated that the proposed method behaved more accurately and stably. We showed the localization results of these datasets in Additional file 2: Figure S1-S9. Notably, for better localization, all the datasets were preprocessed by the method in “Estimation of the soma’s region”, and we used the preprocessed datasets as the input of these methods.

## Discussion

Methods [44, 50, 51] using the typical clustering method mean-shift [52] have been proposed to locate cells. Recently, Frasconi et al. [44] proposed a method for the large-scale localization of somas in which mean-shift was employed to estimate the initial soma positions. As indicated in Fig. 4, it is difficult to find a reasonable parameter when using mean-shift for estimating the positions of somas with a diversity of sizes. Therefore, this method considers the structure of the mouse brain region and regards it as prior information for the identification of the final soma positions. This operation significantly enhances the localization accuracy but constrains its application range. In contrast to this method, our method uses density-peak clustering rather than mean-shift to locate somas, and does not require prior structure information for soma identification. Thus, our method may have a wider application than this method and maintain a high level of localization accuracy.

Generally, the localization of cells consists of three steps: image preprocessing; initial cell localization; and identification of the real initial positions. For example, Frasconi’s method uses supervised semantic deconvolution for image preprocessing, mean-shift to estimate the initial cell positions, and structure information from the brain region to identify the real somas. Methods based on model fitting, including our previous NeuroGPS [43], use the threshold value to extract the soma’s region, search for positions corresponding to signal peaks that are regarded as the initial positions, and model fitting to screen the real soma positions. FarShight method [42] is not an exception, and employs a multi-scale Laplacian of Gaussian (LoG) filter to initially estimate the soma positions. The estimation of the initial positions is an important factor to determine the soma localization accuracy. In contrast to the above methods that use the feature (i.e., the soma center featured by a signal peak), our method introduced density-peak clustering, which allowed the soma center to be featured by a signal peak and a large distance δ between the signal peak point and its closest point corresponding to the higher signal peak. Thus, our method used more features, which might make the estimation of the initial soma positions be more accurate.

Image preprocessing is required for soma localization. The goal of image preprocessing is to increases the signal difference between the soma’s region and the background region, and to decrease the difficulties in extracting the soma’s region. Considering the diversity of images, it is difficult to find one commonly used image preprocessing method that is suitable for most soma images. Our method employs binarization and erosion to extract the soma’s region. The corresponding parameter settings are used to accurately extract the soma’s region based on the following assumptions: 1) the estimated radius of somas ranges from 3 to 10 μm; 2) the background noise is Poisson noise; and 3) the voxel size ranges from 1 × 1 × 1 μm^3^ to 2 × 2 × 2 μm^3^. With the exception of the assumption 3, the assumptions are suitable for the most cell imaging. We also note that the binarization parameter is not relevant to the voxel size, and that the estimation of the soma’s region for a voxel size far smaller than 1 × 1 × 1 μm^3^ can be obtained with high accuracy by increasing the erosion intensity. The above analysis indicate that our image processing method can be applied to most image stacks.

The proposed method contains five main parameters: *thre*_binarization_; *thre*_erosion_; the maximum value of the erosion threshold *T*; Gaussian kernel width σ; and the threshold for selecting candidate cluster center points *thre*_selective_. The preprocessing parameters, *thre*_binarization_, *thre*_erosion_ and the maximum value of the erosion threshold *T* are used to accurately extract the soma regions. The *thre*_binarization_ setting is used to ensure that the extracted foreground can completely cover the soma regions. The erosion operation is used to eliminate the artifacts and noise points in the binarized image stack. *thre*_erosion_ and *T* are used to control the intensity of the erosion, and their settings are determined by the voxel size and the soma radii. We verified that these settings were suitable for voxel sizes ranging from 1 × 1 × 1 μm^3^ to 2 × 2 × 2 μm^3^ and soma radii ranging from 3 μm to 10 μm, indicating that these settings were suitable for most cell images. The Gaussian kernel width σ is a scale parameter used to compute the local density and is related to the soma radii. Too larger values of σ usually lead to under-segmentation, whereas too small values may result in over-segmentation. A suitable σ value is about half of the average value of the soma radius (3 – 4 μm). The threshold *thre*_selective_ is used to select candidate cluster center points. Cluster centers are characterized by a higher density ρ than their neighbors and by a relatively large distance δ, and act as isolated points in the ρ-δ feature space. Therefore, the possible cluster center points can be selected according to the feature density Λ (the density computed in the ρ-δ feature space). The *thre*_selective_ setting should ensure that all soma positions are included in the candidate cluster center points. Therefore we set it to a small value of 10^-2^ in our experiment. This setting is suitable for diverse datasets, including two-photon fluorescence datasets [16], Nissl staining datasets [46], structured illumination microscopy datasets [47], and wide field fluorescence datasets [54]. In short, for different datasets, clustering parameters are easily set, and we usually need to set suitable preprocessing parameters for accurately extracting soma regions according to the image contrast, the influence of neurites. For datasets with many neurites (such as Data 1 – 5 in Table 1), we need to fine tune the preprocessing parameters to eliminate the neurites and keep soma regions. In this case, the proposed method can be categorized as a semi-automated approach. For datasets without neurites (such as Data 6 – 9 in Table 1), we only need to set the binarization parameter according to the image contrast and the complex erosion operation is not necessary. Therefore, the proposed method can work relatively automatically.

## Conclusions

In conclusion, we propose a novel method for the localization of touching somas based on modified density-peak clustering. This method can effectively locate somas from large-scale and complicated datasets. Furthermore, we have demonstrated the strong robustness of the key parameter for the localization and its effectiveness at a low SNR level. Thus, the method provides an effective tool for the neuroscience community to quantify the spatial distribution of neurons and the morphologies of somas.

## Additional files

Additional file 1: Detailed derivation of the proposed method complexity. (PDF 104 kb) Additional file 2: The localization result comparison of the proposed method and other methods on nine different datasets. (PDF 7671 kb)

## Abbreviations

2p-fMOST: Two-photon fluorescence micro-optical sectioning tomography system
GFT: Gradient flow tracking
SNR: Signal-to-noise ratio
