# Supplementary material for: Large-scale localization of touching somas from 3D images using density-peak clustering
Source: BMC Bioinformatics. 2016 Sep 15;17:375. doi: 10.1186/s12859-016-1252-x (PMC5024436; doi:10.1186/s12859-016-1252-x)
Supplement: Additional file 1: — Detailed derivation of the proposed method complexity. (PDF 104 kb) [file 12859_2016_1252_MOESM1_ESM.pdf]

Additional File 1: Detail derivation of the proposed method complexity

The localization part of our method includes three steps: computing local density ρ, searching the minimum distance δ, identifying cluster center points. The bottleneck is the search for the minimum distance. We reduce the searching space to speed up our method using two strategies: locating somas in every connected region rather than in the whole image stack and using local search for the minimum distance in each connected region. We demonstrated that the complexity of our method is about proportional to the volume of the image stack. Below is the detailed description of our method.

Since we locate somas in each connected region rather than the whole image stack, the complexity of our localization method is about proportional to the complexity of finding soma positions in one connected region. The proportional coefficient is approximately equal to the number of the connected regions. In the following, we focus on deriving the complexity of one connected region. Let I_i be the minimum block containing the *i*th connected region. Let n_i *be* the number of the foreground voxels in I_i.

Computing local density ρ:

$$\rho = \frac{1}{Z}\sum_{q:\|p-q\|_2 \le R} I(q)K(p,q) = \frac{1}{Z}\sum_{q:\|p-q\|_2 \le R} I(q)\frac{1}{\sqrt{2\pi}\sigma}\exp(-\frac{\|p-q\|_2^2}{2\sigma^2})$$

p, *q* are foreground voxel points, *R* is the window radius of kernel function. According to the above density computation formula, the complexity of computing the local density of one foreground voxel is $O(R^3)$. Therefore, the complexity of the *i*th connected region is $O(n_i R^3)$.

Searching the minimum distance δ:

$$\delta = \begin{cases} \dfrac{\min\limits_{q:\rho_q > \rho_p} \|p-q\|_2}{\max\limits_{\forall p,q} \|p-q\|_2} & \rho_p < \max\limits_{\forall q} \rho_q \\ 1 & \rho_p = \max\limits_{\forall q} \rho_q \end{cases}$$

To search the minimum distance of one foreground voxel, we need to compute the

minimum values n_i - *1* times from n_i - 1 values, n_i - 2 values, n_i - 3 values, …, 1 value. As we know the complexity of finding the minimum value in an unsorted array with n values is O(n), so the complexity of searching the minimum distance of one foreground voxel is below O(n_i^2). We take the local searching strategy (11 × 11 × 11 voxel neighboring region) rather than global searching when the number of the foreground voxels in a connected region is more than 3000. (If we cannot find the desired minimum distance in the local searching, we can take the current point as an candidate cluster center since the maximum radius of somas is limited, about 10 μm 5 voxel. Thus, the local searching does not influence the localization.) As a consequence, the complexity in this step is O($11^3 n_i$) (n_i > 3000) or O(n_i^2) (n_i <= 3000), and is always less than O(3000n_i).

Identifying cluster center points:

In this step, the main computation is in computing the feature density Λ, and is constant, about 10^8 times multiplication.

Therefore, the complexity of one connected region is O($n_i R^3$) + O(3000n_i), and the complexity of all connected regions is Σ [O($n_i R^3$) + O(3000n_i)]. Here, R is the window radius of kernel function, n_i is the number of the foreground voxels in one connected region and is usually less than 3000, and the number of the connected regions is about proportional to the volume of the image stack. As a consequence, the complexity of our method is about proportional to the volume of the image stack as showed in Fig. 4c of the manuscript.
