# Supplementary material for: Large-scale localization of touching somas from 3D images using density-peak clustering
Source: BMC Bioinformatics. 2016 Sep 15;17:375. doi: 10.1186/s12859-016-1252-x (PMC5024436; doi:10.1186/s12859-016-1252-x)
Supplement: Additional file 2: — The localization result comparison of the proposed method and other methods on nine different datasets. (PDF 7671 kb) [file 12859_2016_1252_MOESM2_ESM.pdf]

Additional File 2: The localization result comparison of the proposed method and other methods on nine different datasets

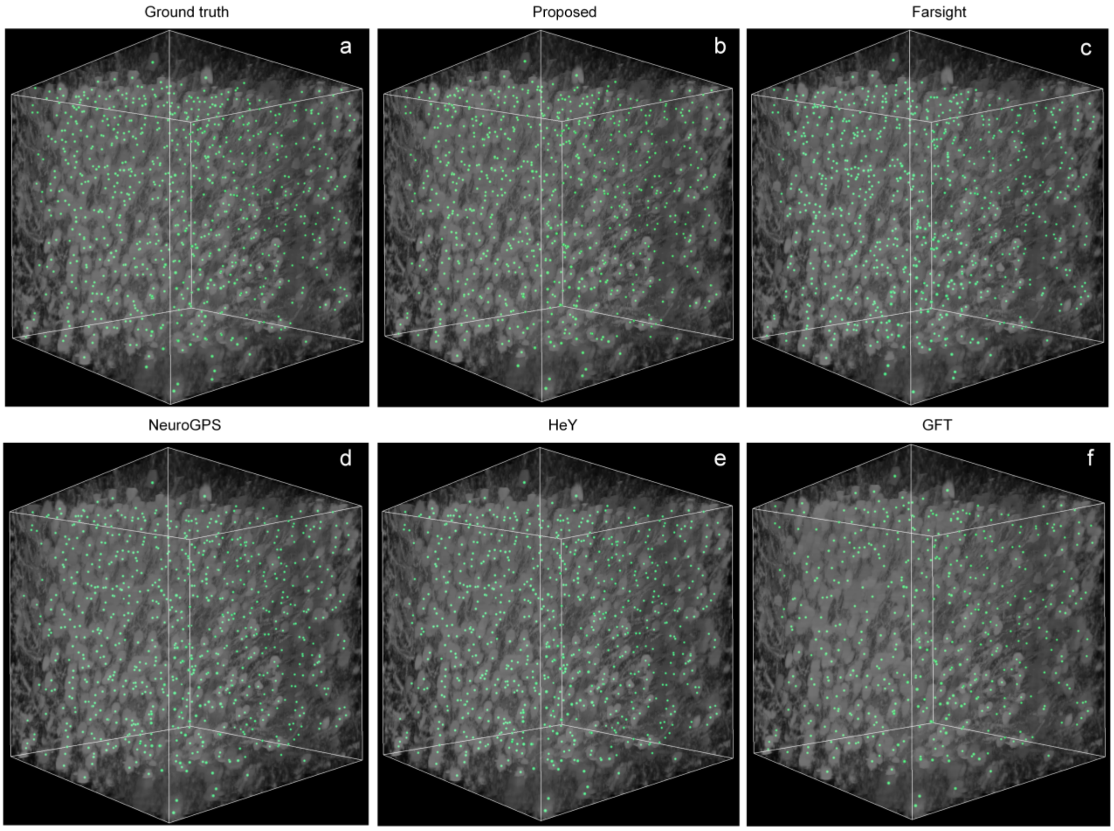

Fig. S1. Localization results of Data1.

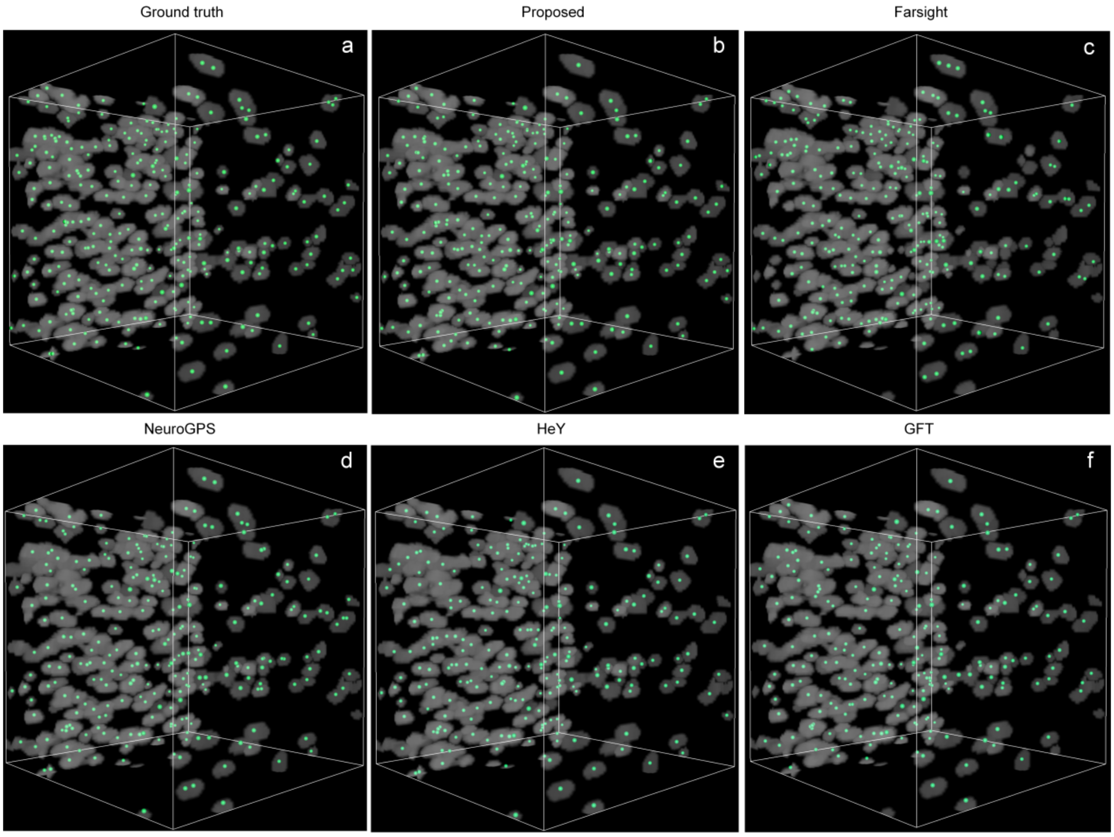

Fig. S2. Localization results of Data2. The preprocessed images are shown.

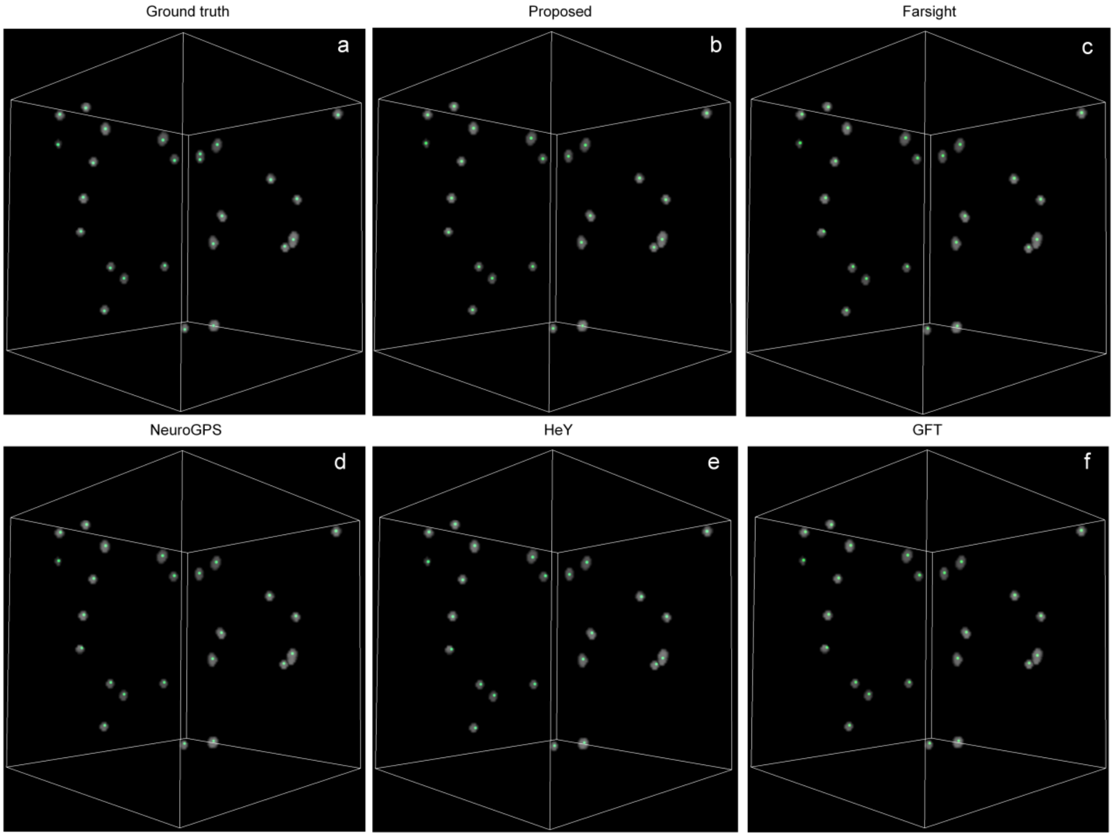

Fig. S3. Localization results of Data3. The preprocessed images are shown.

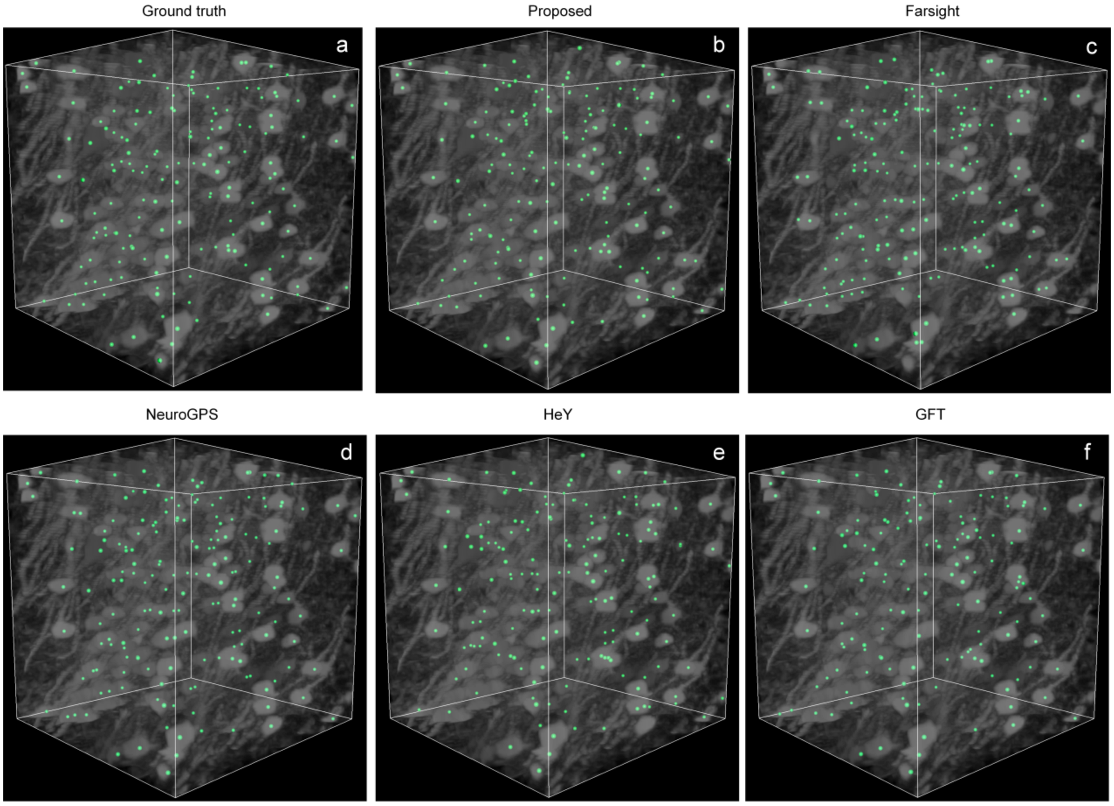

Fig. S4. Localization results of Data4.

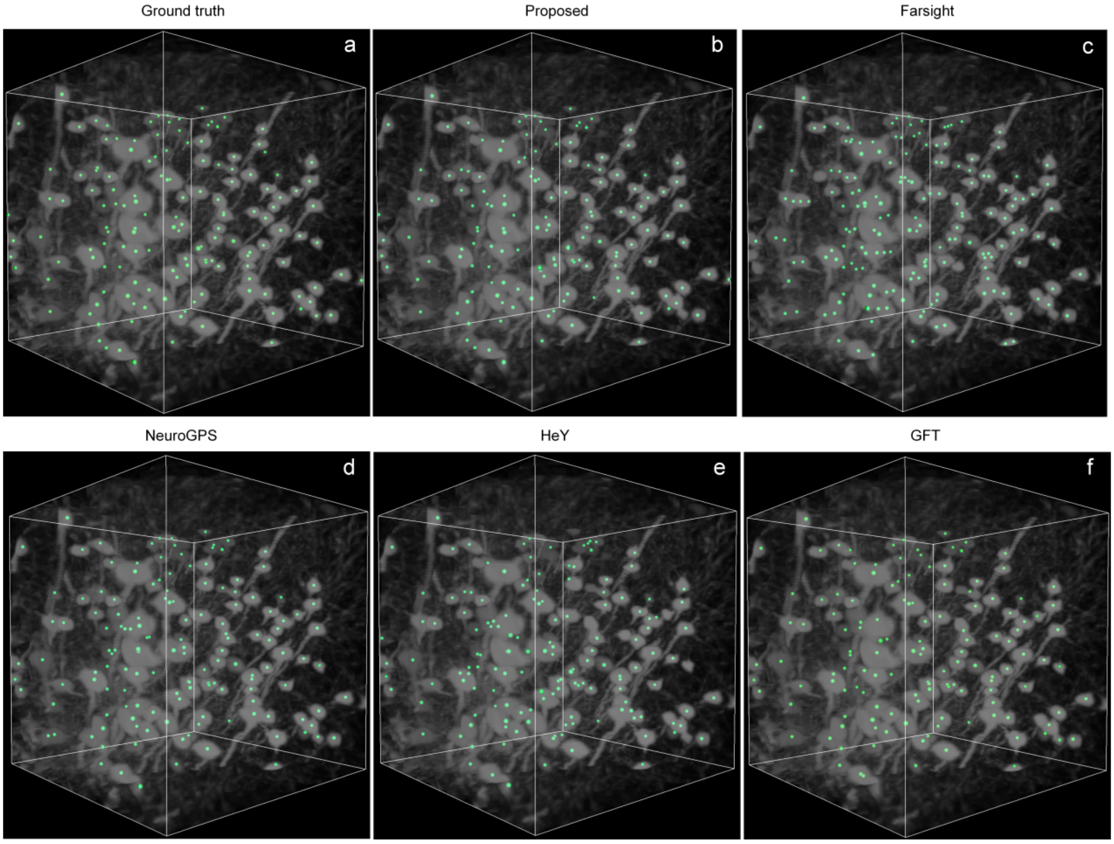

Fig. S5. Localization results of Data5.

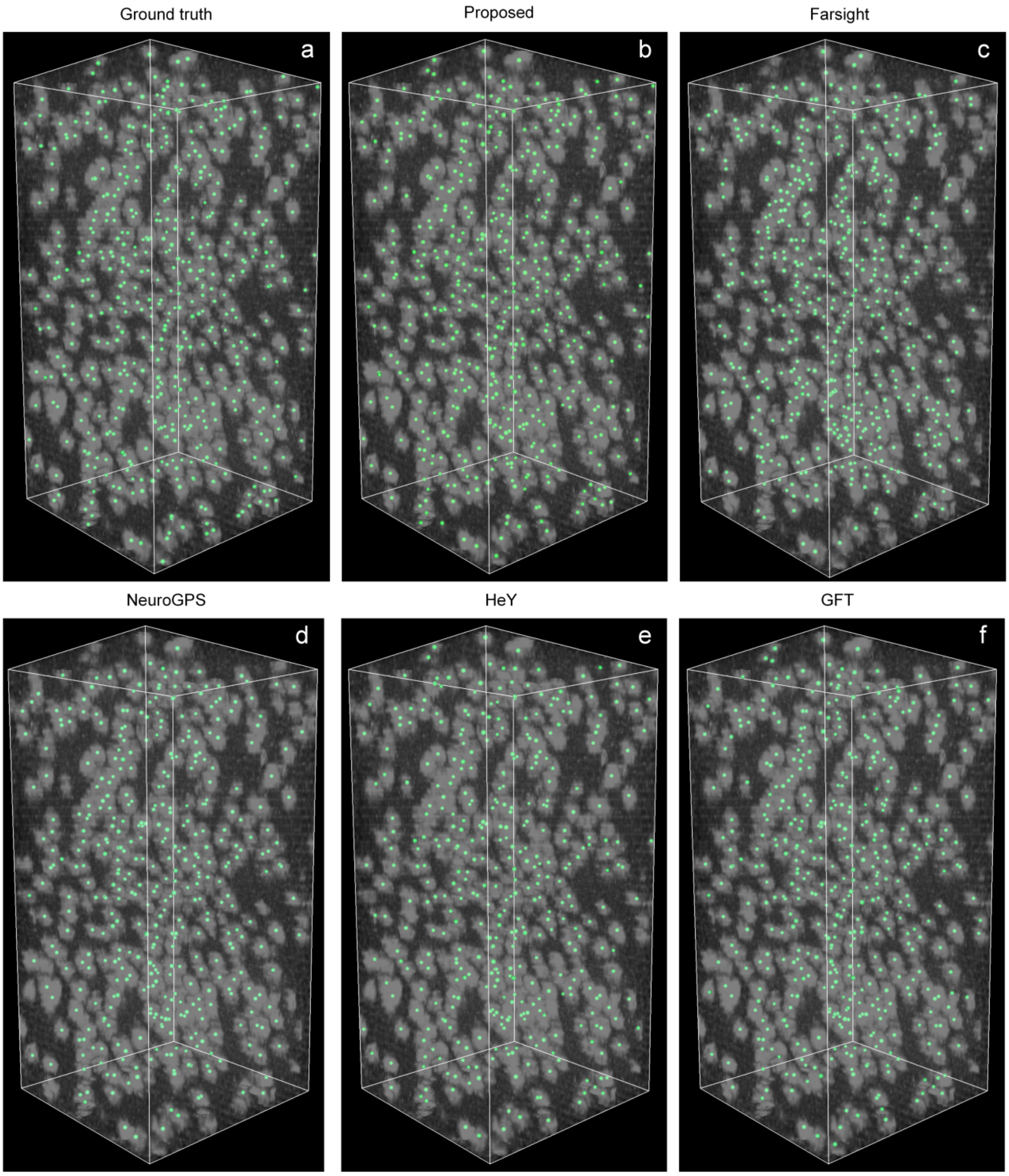

Fig. S6. Localization results of Data6.

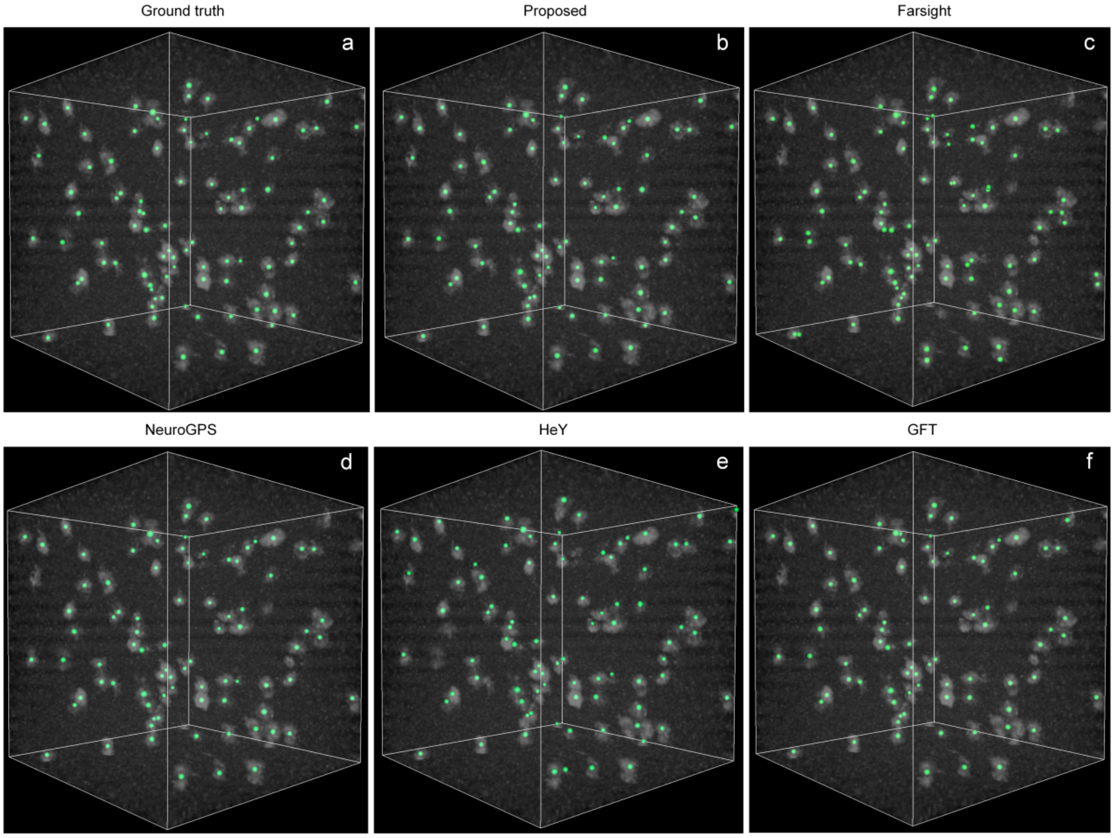

Fig. S7. Localization results of Data7.

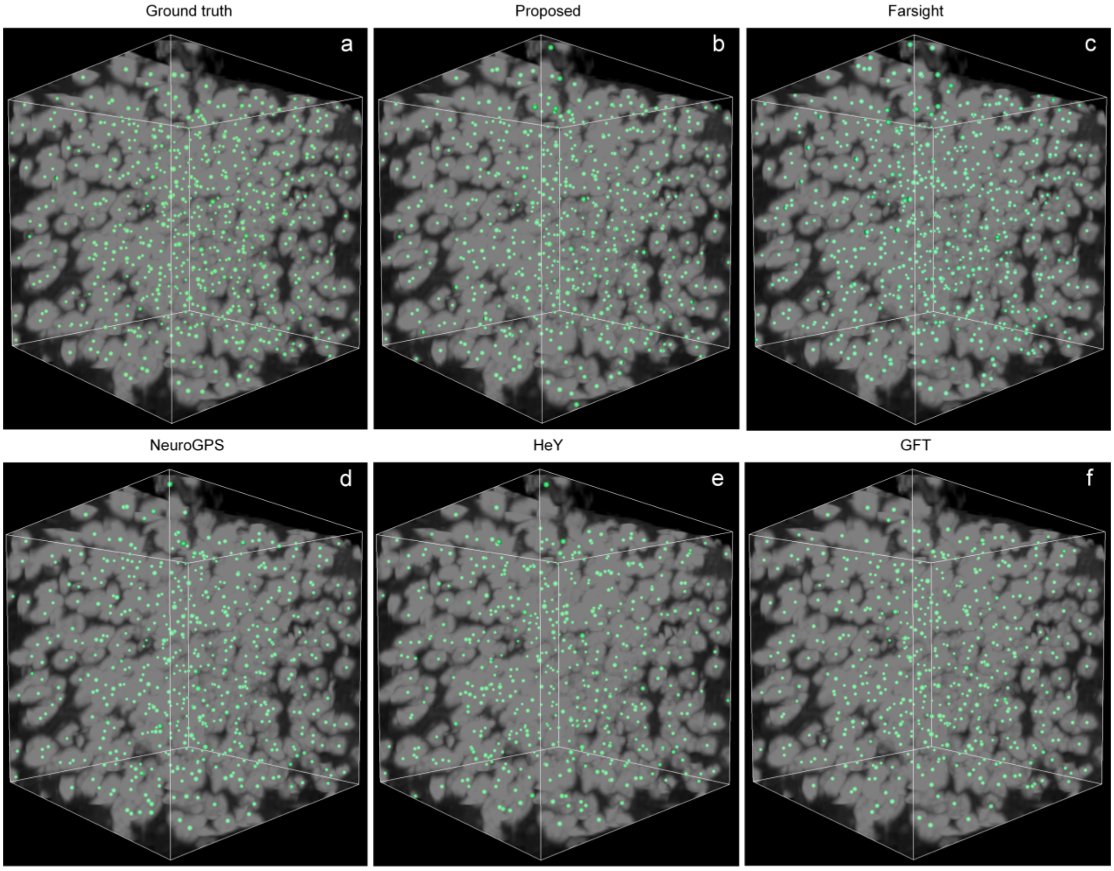

Fig. S8. Localization results of Data8.

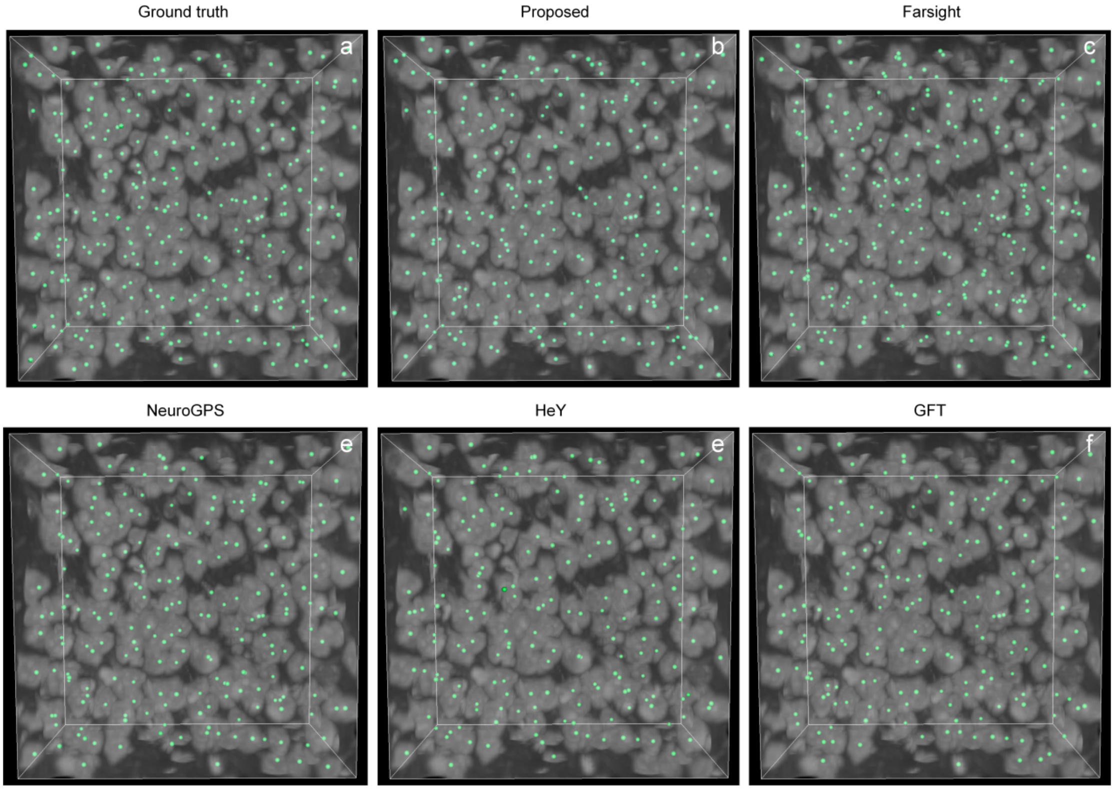

Fig. S9. Localization results of Data9.
